# Analysis of Factors Affecting Depression in Older Adults in South Korea

**DOI:** 10.3390/ijerph18189887

**Published:** 2021-09-20

**Authors:** Ah-Ram Kim, Jin-Hyuck Park, Hae Yean Park

**Affiliations:** 1 Department of Occupational Therapy, Graduate School, Yonsei University, Wonju 26493, Korea; aramkim495@gmail.com; 2 Department of Occupational Therapy, Soonchunhyang University, Asan 31538, Korea; roophy@naver.com; 3 Department of Occupational Therapy, College of Software and Digital Healthcare Convergence, Yonsei University, Wonju 26493, Korea

**Keywords:** depression, Korean Longitudinal Study of Aging (KLoSA), machine learning, older adult

## Abstract

**Objective:** This study aimed to analyze the factors affecting depression among South Korean middle-aged and older adults using data from the Korean Longitudinal Study of Aging. **Methods:** We analyzed data regarding demographic characteristics, lifestyle, quality of life, cognitive level, and depression. Cognitive level and depression were evaluated using the Korean-Mini-Mental Status Examination and Center for Epidemiological Studies Depression Scale, respectively. **Results:** Depression was correlated with age, gender, residential area, level of education, alcohol intake, regular exercise, life satisfaction, and cognitive level, but not smoking. Furthermore, depression was highly affected by age, residential area, regular exercise, life satisfaction, and cognitive level, with a prediction accuracy of 80.26% achieved through machine learning analysis. **Conclusions:** Various factors are associated with depression in middle-aged and older adults. Therefore, multifaceted interventions for preventing depression in these age groups are required.

## 1. Introduction

South Korea is experiencing an increasingly aged population, with 15.8% of the population aged ≥65 years as of 2020 [1]. Currently, the average life expectancy of South Koreans is 83.2 years (2020), which is expected to rise to 86.8 years after two decades [2]. In 2019, the life satisfaction of South Koreans according to age group was found to be lowest among individuals in their 60s (the scores out of 10 points for every age group were as follows: teens and 20s, 6.0 points; 30s, 6.1 points; 40s, 5.9 points; 50s, 6.1 points; and 60s, 5.8 points) [3]. A study on happiness by life cycle in Japan and South Korea that used data from the East Asian Social Survey reported a negative correlation between happiness and age [4]. South Korea is about to become a super-aged society. Therefore, there is a need to actively seek intervention measures for improving the quality of life in older adults at the social level and to focus on providing necessary resources.

Various factors affect the quality of life or satisfaction in older adults. Life satisfaction is not an objective condition; rather, it is a combination of an internal and subjective process [5]. Therefore, there has been growing interest in factors associated with life satisfaction, especially psychological or personality factors, including depression, anxiety, and neurosis [6]. Among the psychological factors, depression has negative effects, including increasing the socioeconomic burden, reducing the quality of life among older adults, and increasing the rates of morbidity and mortality [7].

Depression is a universal human emotion characterized by a continuous variation that ranges from mild to abnormal levels [8]. However, depression is more common in late stages of life, with 20–25% of older adults experiencing depression [9]; this decreases cognition and the overall quality of life [10] and is associated with the overall risk of death. Therefore, there is a need to identify the risk factors for depression and to establish prevention measures to improve health and independence in older adults [11]. 

Previously reported factors that are associated with depression in older adults include sex, age, level of education, unfavorable lifestyles (e.g., smoking and excessive alcohol consumption), and psychological stress [10,12]. Further, regular exercise is a crucial factor in the prevention and treatment of depression. Regular exercise reduces depression in healthy adults, as well as in patients with diabetes, stroke, or cancer [12]; further, older adult individuals who actively exercise are less likely to be depressed than those who do not exercise [12,13]. Additionally, exercising can effectively treat depression and reduce the use of antidepressants [14]. Although there have been studies on the association of quality of life with physical activity and health in South Korea [15], few relate to depression. Therefore, there is a need to elucidate the influence of physical activity on factors affecting depression among older adults.

To overcome the limitations of previous studies, this study aimed to employ a machine learning approach to analyze large-scale longitudinal data obtained from the Korean Longitudinal Study of Aging (KLoSA). Machine learning is an artificial intelligence field involving statistical models and algorithms that allow machines to self-train and to perform specific tasks without commanding code for each factor [16]. It employs large-scale data and is divided into supervised and unsupervised learning. Supervised learning involves learning collected data for future prediction whereas unsupervised learning seeks to discover hidden patterns or unique structures within input data. We can use a disambiguation model algorithm employing supervised learning as a basis for providing universal support for the entire population’s happiness by discovering predictive variables for depression among middle-aged (age: 55–64 years) and older adults (age: ≥65 years). This study aimed to compare the accuracy between the predictive model and main predictive variables for depression using KLoSA data obtained from middle-aged and older adults.

## 2. Material and Methods

### 2.1. Participants and Data Analysis

The KLoSA has been sampling and surveying middle-aged and older adults aged ≥ 45 years living in areas other than Jeju Island since 2006, with the 7th basic investigation being completed in 2018 [17]. This study was conducted using computer-assisted personal interviewing. We analyzed the KLoSA data obtained in 2018 from 6940 participants. In this study, participants 55 years of age or older were selected as the older adults. In general, the standard for the elderly is set at 65 years of age or older, but in previous studies related to the elderly, including the National Informatization White Paper, those in their 50s or older are set as one older adult group [18]. Data were collected from those who voluntarily agreed to participate in the research in advance. This study was conducted after obtaining approval from Yonsei University’s Institutional Review Board in accordance with research ethics (Approval number: 1041849-202012-SB-187-01).

### 2.2. Factors

#### 2.2.1. Personal Factors

Personal factors included sex, age, religion, residence area, and level of education. Sex was divided into male and female, while age was calculated by subtracting the year of birth from 2018. Religion was classified as “religious” or “not religious,” while residential areas were classified into large, small, and medium cities, and towns/villages. The level of education was classified as elementary, lower, middle, and high school, as well as college.

#### 2.2.2. Lifestyle

Lifestyle factors included alcohol consumption, smoking, and regular exercise. Drinking and smoking were classified into yes or no based on their responses to the questions of whether the participants occasionally drank or smoked, respectively. Regular exercise was determined by asking whether the participants exercised at least once weekly.

#### 2.2.3. Depression

Depression was assessed using the Korean version of the Center for Epidemiologic Studies Depression Scale. It has a cut-off value of 3 points, with a score of 0–2 and 3–10 points being classified as normal and depression, respectively. The questionnaire comprised ten questions regarding feelings and actions from the previous week; moreover, its score is positively correlated with the level of depression [19].

#### 2.2.4. Life Satisfaction

Life satisfaction was scored based on the answer to the question “How satisfied are you with your overall quality of life compared with other similarly aged people?” The score ranges from 0 to 100, with the measurement unit being 10 points. The score is positively correlated with the level of life satisfaction.

#### 2.2.5. Level of Cognition

The level of cognition was assessed using the Korean Mini-Mental State Examination (K-MMSE). K-MMSE is an evaluation tool developed to quantitatively evaluate cognitive impairment [20]. It comprises 30 questions in total. Individuals scoring >24 points are considered normal, those with 18–23 points are considered to have cognitive decline, and those with <17 points are suspected to have dementia [21]. The concurrent validity was 0.660–0.776, and the test-retest reliability was 0.887 [20].

### 2.3. Analysis Methods

We performed frequency analysis with weighing to examine the general characteristics of the study participants. We performed a correlation analysis to determine the simple between-variable correlation and multicollinearity between independent variables. Logistic regression analysis was performed to identify predictor variables associated with the presence or absence of depression, which were manipulated as nominal variables. Machine learning proceeded in three steps. The first step included the data processing step of standardizing basic variables. When logistic regression analysis is used for machine learning, data are normalized to prevent data divergence. The second step was to randomly divide the data into a training dataset and a test dataset, and each set was developed to generate feature (x_data) and outcome (y_data) variables. The third step included model training with the train dataset via a logistic regression model using TensorFlow (Google Brain, State of California, United States of America. The machine learning was conducted until the cost value was stabilized, and the number of learning steps was 10,000. Machine learning analysis was validated using the hold-out method with the ratio of training and test data being maintained at 10:1. Moreover, it was used to confirm the accuracy of predictive factors identified by regression analysis.

## 3. Results

### 3.1. Demographic Characteristics

The 7th year KLoSA sample comprised data from 6940 people; among them, 6064 individuals were analyzed in the present study (Table 1). The included sample comprised 2600 (42.88%) males and 3464 (57.12%) females. Regarding age groups, most of the individuals (2468 [37.70%]) were in the 55–64-years group, followed by the 65–74 group and the 75–84 group. Most of the participants lived in large cities (40.30%), followed by small and medium cities (32.68%), and towns/villages (24.01%). Regarding the level of education, most of the participants (2129 [35.11%]) had elementary or lower education, followed by high school graduates, middle school graduates, and those with a college degree or higher education. Further, 65.27% and 89.99% of the respondents reported that they did not drink alcohol or smoke, respectively. Additionally, 65.27% of the participants did not exercise regularly and most respondents scored their life satisfaction with scores of 40–70. Regarding the level of cognition, 4584 (75.59%), 1056 (17.41%), and 424 (6.99%) individuals presented with normal cognitive level, cognitive function decline, and suspected dementia, respectively. Finally, in terms of the presence or absence of depression, 3236 (53.36%) individuals were considered normal and 2828 (46.64%) were considered depressed. 

### 3.2. Correlation between Depression and the Analyzed Factors

All investigated factors, apart from smoking, were correlated with depression. Specifically, age (0.192, *p* = 0.000), sex (0.062, *p* = 0.000), residential area (0.060, *p* = 0.000), level of education (−0.153, *p* = 0.000), and alcohol consumption (−0.089, *p* = 0.000) were positively correlated with depression (Table 2). Moreover, older age, being female, lower level of education, and no alcohol consumption were associated with higher depression levels. On the other hand, regular exercise (0.083, *p* = 0.000), life satisfaction (−0.286, *p* = 0.000), and K-MMSE score (−0.286, *p* = 0.000) were negatively correlated with depression. There was no significant correlation between smoking and depression (−0.018, *p* = 0.168). In other words, the less regular exercise, the lower the life satisfaction, and the higher the cognitive level were associated with higher depression levels.

### 3.3. Effects of the Factors on Depression

Logistic regression analysis was performed to determine the effects on depression. Before regression analysis, we performed a correlation analysis to identify factors associated with depression (Table 3). Regression analysis revealed that age (*B* = 0.018, *p* < 0.001), residential area (*B* = 0.079, *p* < 0.05), final educational level (*B* = 0.069, *p* < 0.05), regular exercise (*B* = 0.030, *p* < 0.05), life satisfaction (*B* = −0.032, *p* < 0.001), and MMSE score (*B* = −0.096, *p* < 0.001) all had significant effects on depression. Specifically, the depression risk increased by a factor of 1.019, 1.082, 1.072, 1.030, 0.969, and 0.909 as age increased, when living in a city with a small population, with lower levels of education, without regular exercise, as life satisfaction decreased, and as cognitive level decreased, respectively. Particularly, the residential area showed the highest increase rate with the Exp(*B*) value indicative of change. The explanatory power (R2) of the independent variable for depression was 13.08%. The 2 log-likelihood and chi-square (x2) values for confirming the model’s fit were significant at 7507.845 and 871.172, respectively (*p* < 0.001), which indicated that the model was suitable.

### 3.4. Accuracy of the Factors Affecting Depression

Machine learning was applied to evaluate the accuracy of factors affecting depression through regression analysis. Data from 5893 and 654 participants were applied as the training and test data, respectively. We analyzed the accuracy of CASE 1, trained with residential area, final educational level, and regular exercise; CASE 2, trained with age, MMSE score, and life of satisfaction; CASE 3, trained with age, residential area, final educational level, regular exercise, and life of satisfaction; CASE 4, trained with age, residential area, final educational level, regular exercise, and MMSE score; and CASE 5, trained with life of satisfaction in addition to the training for CASE 4. Table 4 shows the results after the comparisons of each factor. Comparison of the training and test data revealed accuracy of 55.6%, 64.4%, 64.2%, 58.6%, and 78.2%, respectively.

## 4. Discussion

In this study, depression was correlated with age, sex, residential area, and level of education. Previous local and international studies on depression in older adults have reported that being female [22,23], being older [22], having a lower level of education [24], and being an unhealthy older adult [23] as the major factors affecting depression. According to previous studies in Korea, depression was lower for the older adults living in areas closer to a metropolis [25]. This is thought to be because more diverse housing types, convenient commercial districts, and transportation are located near the city center, making it easier to access local communities. In this study, regression analysis revealed that the residents of towns and villages were more likely to be depressed. Regarding the residential area, there have been inconsistent reports that older adult individuals in rural areas are less [23] or more prone to depression [11]. Further, regarding machine learning, CASE 4 differed from CASE 3 in that it included residential area in the training step; however, there were no differences in accuracy. Therefore, there is a need for further studies on depression based on residential area.

Regarding health-related factors, alcohol consumption and regular exercise were correlated with depression, which is consistent with previous reports of a positive correlation between alcohol consumption and depression levels [26]. Alcohol is responsible for 10% of all dementia cases [27]; moreover, heavy drinking negatively affects cognitive health in older adults [28]. Therefore, there is a need to address alcohol consumption among older adults since it may result not only in physical or health-related issues but also in more severe secondary issues. Our findings revealed a significant correlation of regular exercise with depression. This is consistent with a previous report that in older adults, regular exercise and physical activity positively affects mental health while strength training reduces depression [29].

We did not observe a correlation between smoking and depression, which is inconsistent with previous findings [30]. This could be attributed to the low number of smokers (9.44%) in our study. There is a need for future studies with more smokers to assess the correlation between depression and smoking. Depression was found to affect life satisfaction among older adults, which is consistent with a previous report [31]. Moreover, depression has been shown to decrease life satisfaction and is the most influential factor in suicide attempts [32]. Therefore, the degree of depression markedly influences life satisfaction. According to a prior study, participating in social activities increases the satisfaction of the elderly and helps maintain a positive attitude toward life [33].

We also found a correlation between depression and level of cognition. Numerous studies have assessed the correlation between depression and cognitive decline; however, the causal and biological mechanisms remain unclear [34]. The increased depression among patients with cognitive impairment remains unexplained; however, it could be attributed to the psychological distress caused by a decrease in daily life ability or social activity secondary to cognitive decline [35]. Given the strong association between cognitive decline and depression, there is a need to maintain cognitive health for preventing depression.

To compare the accuracy of factors identified by regression analysis, we included the three factors with highly significant results (*p* < 0.001; age, life satisfaction, and cognitive level) and the six factors with significant results (*p* < 0.05; age, final educational level, residential area, regular exercise, life satisfaction, and cognitive level) in the comparison. The accuracy of CASE 1 was 55.6%, and that of CASE 5 was 78.2%. This is indicative of not only the factors affecting depression in older adults but also the complex mechanisms underlying them. To promote healthy lives among older adults in an aging society, there is a need for interventions that promote health and prevent depression, as well as to prevent chronic diseases and depression by altering lifestyles harmful to health [36]. Given the close correlation between lifestyle and the quality of life of older adults, their lifestyles should be evaluated from multiple perspectives [37] and multifaceted interventions are likely required to prevent depression.

For preventing depression and improving health and quality of life of the older adults, there is a lifestyle intervention program with multifaceted approaches to improve the lives of older adults. Maintaining a healthy lifestyle, including nutrition, physical activity, cognitive and social participation, and metabolic and vascular risk monitoring are known to be important in reducing the risk of cognitive decline and disability [38]. It is also reported that the intervention program, including these lifestyle factors, promoted balanced nutrition habits, exercise, and social participation of older adults, improving their quality of life [39]. Lifestyle intervention is increasingly emerging as an important goal for preventing dementia and depression, promoting health in the older adults [40]. Therefore, in order to reduce depression and improve health of the older adults, research on various intervention methods that adopt this healthy lifestyle is needed.

This study is not without limitations. First, this study only used KLoSA data obtained from middle-aged (55–64 years) or older adults (≥65 years). Future studies should fully utilize the longitudinal data of the middle-aged and older adult panels to analyze factors influencing depression according to life period. Second, our machine learning model only compared the magnitude of importance of each variable and could not predict the inter-relationships among the variables. Further studies are required to analyze the relationship between the major variables identified in this study using a model with various structures. Third, this study only analyzed data and variables obtained from the KLoSA. Future studies employing additional data and various variables could allow the identification of more accurate and multifaceted predictor variables.

Nonetheless, this study is significant in that it used machine learning analysis rather than traditional analysis methods, using actual big data accumulated using relatively controlled surveys of the KLoSA. There have been rare occupational therapy studies applying machine learning techniques using big data. Actual big data tend to be complicated by missing or incorrectly recorded information. Since machine learning techniques can more comprehensively handle these problems than traditional linear regression and other statistical methods, they could be advantageous for future related studies, with this study providing a meaningful first step in that direction.

## 5. Conclusions

This study identified factors affecting depression in the older adults using data obtained in the 7th KLoSA. Depression was correlated with age, gender, residential area, level of education, drinking, regular exercise, life satisfaction, and cognitive level, but not smoking. Moreover, depression was highly affected by age, residential area, regular exercise, life satisfaction, and cognitive level, with an accuracy of 80.26% confirmed by machine learning. There is a need for prospective studies to more clearly elucidate the relationship between depression and cognition. Additionally, in order to reduce depression and improve the health of the older adults, research on various intervention methods that can adopt this healthy lifestyle is needed.

## Figures and Tables

**Table 1:** Demographic characteristics (*N* = 6064).

			*N*	%
Personal	Sex	Male	2600	42.88
		Female	3464	57.12
	Age	55–64	2468	40.70
		65–74	1937	31.94
		75–84	1659	27.36
	Residential area	Metropolis	2626	43.30
		Small and medium-sized city	1982	32.68
		Town and village	1456	24.01
	Educational level	Below the elementary school	2129	35.11
		Middle school	1058	17.45
		High school	2077	34.25
		College graduate or higher	800	13.19
Lifestyle	Drinking	Yes	2100	34.63
		No	3964	65.37
	Smoking	Yes	607	10.01
		No	5457	89.99
	Regular exercise	Yes	2106	34.73
		No	3958	65.27
Life of satisfaction		0–30	383	6.32
		40–70	4241	69.94
		80–100	1440	23.75
Cognition level		Suspected dementia	424	6.99
		Cognitive decline	1056	17.41
		Normal	4584	75.59
Depression		Normal	3236	53.36
		Depression	2828	46.64

**Table 2:** Correlation between depression and factors.

		Correlation Coefficient	*p*-Value
Personal	Age	0.192 ***	0.000
	Sex	0.062 ***	0.000
	Residential area	0.060 ***	0.000
	Final educational level	−0.153 ***	0.000
Lifestyle	Drinking	0.089 ***	0.000
	Smoking	−0.018	0.168
	Regular exercise	−0.083 ***	0.000
Life of satisfaction		−0.286 ***	0.000
MMSE		−0.286 ***	0.000

*** *p* < 0.001

**Table 3:** Effects of the different factors on depression.

		*B*	Exp(*B*)	*p*-Value
Personal	Age	0.018 ***	1.019	0.000
	Sex	0.031	1.032	0.051
	Residential area	0.079 *	1.082	0.029
	Final educational level	0.069 *	1.072	0.033
Lifestyle	Drinking	−0.053	0.949	0.415
	Regular exercise	0.030 *	1.030	0.046
Life of satisfaction		−0.032 ***	0.969	0.000
MMSE		−0.096 ***	0.909	0.000
Adjust R^2^		13.08		
−2 log likelihood		7507.845		
Model x^2^		871.172 ***		

*** *p* < 0.001, * *p* < 0.05.

**Table 4:** Accuracy of the factors affecting depression.

	CASE 1	CASE 2	CASE 3	CASE 4	CASE 5
Factor	Residential area	Age	Age	Age	Age
	Final educational level	MMSE	Residential area	Residential area	Residential area
	Regular exercise	Life of satisfaction	Final educational level	Final educational level	Final educational level
			Regular exercise	Regular exercise	Regular exercise
			Life of satisfaction	MMSE	Life of satisfaction
					MMSE
Training data (*n*)	5000				
Test data (*n*)	500				
Max iteration	10,000				
Momentum	0.5				
Accuracy (%)	55.6	64.4	62.4	58.6	78.2

## Data Availability

The data used in this analysis from the Korea Employment Information Service and are available on its web page http://survey.keis.or.kr (accessed on 7 October 2020).
